# Microindentation hardness and calcium/phosphorus ratio of dentin following excavation of dental caries lesions with different techniques

**DOI:** 10.1186/s40064-016-3289-8

**Published:** 2016-09-22

**Authors:** Gunseli Katirci, R. Banu Ermis

**Affiliations:** Department of Restorative Dentistry, Faculty of Dentistry, Suleyman Demirel University, 32260 Isparta, Turkey

**Keywords:** Dental caries, Dental cavity preparation, Energy dispersive X-ray spectroscopy, Er:YAG lasers, Hardness, Scanning electron microscopy

## Abstract

**Background:**

The aim of this in vitro study was to evaluate the microindentation hardness and chemical composition of residual dentin left at the cavity bottom following removal of carious dentin using the Carisolv chemomechanical and Er:YAG laser caries excavation methods in comparison with the conventional tungsten-carbide bur excavation.

**Methods:**

Sixty-nine extracted permanent teeth with occlusal dentin caries were assigned into three groups according to caries removal technique. Carious dentin excavation was guided by tactile method and a caries-staining dye. In stereomicroscope images (100×) of the samples, the presence or absence of residual caries was defined. The Knoop hardness value of the cavity floor was determined and atomic analysis of treated cavities was performed by energy dispersive X-ray spectroscopy.

**Results:**

The Knoop hardness value of residual dentin left at the cavity bottom was lower (One-way ANOVA, Dunnett-C, p < 0.05) and the percentage of samples with remaining carious dentin was higher after Carisolv excavation than those obtained after conventional and laser excavations (Kruskal–Wallis, Mann–Whitney U, p < 0.05). No significant differences were found between the quantities of calcium content (Ca wt%), phosphorus content (P wt%) and calcium/phosphorus ratio of the cavities treated by three techniques (Kruskal–Wallis, Mann–Whitney U, p > 0.05).

**Conclusion:**

The results indicated that Er:YAG laser was more comparable to conventional bur excavation than chemomechanical method in the efficacy of caries removal with regard to microindentation hardness of remaining dentin and both Carisolv gel and Er:YAG laser did not alter chemical composition of residual dentin in the treated cavities.

## Background

Caries excavation has conventionally been performed using mechanical rotary instruments (handpieces and burs) but alternative techniques such as the use of chemomechanical caries removing agents, sono/air abrasion and laser ablation are presently available today (Banerjee et al. 2000; de Almeida Neves et al. 2011). The rationale of development of the different techniques for caries removal and cavity preparation is to meet the modern concept of minimally invasive dentistry. The objectives of more conservative approaches are to complete removal of carious dentin in order to provide adequate retention for the restorative material producing long-term successful restoration and identification of a superficial infected dentin and a subjacent affected dentin layer being minimally invasive during caries removal (Banerjee et al. 2000; de Almeida Neves et al. 2011).

Chemomechanical caries removing agents are classified as sodium hypochlorite-based agents and enzymatic-based agents (de Almeida Neves et al. 2011; Hamama et al. 2013). Carisolv system (MediTeam Dental, Sweden) mainly contains sodium hypochlorite, three amino acids (glutamic acid, leucine, lysine) and water and has been developed with the purpose of removing all the infected dentin preventing the removal of affected dentin, and is intended to provide less painful caries excavation (Banerjee et al. 2000; de Almeida Neves et al. 2011; Hamama et al. 2013). Sodium hypochlorite has a non-specific proteolytic effect which dissolves the denatured collagen as a result of the action of chlorine. The complete caries removal rate and chemical composition and microstructure of dentin after Carisolv treatment does not seem to be significantly different from conventional rotary instruments (Banerjee et al. 2000; Hossain et al. 2003; Sakoolnamarka et al. 2005; Ramamoorthi et al. 2013; Garcia-Contreras et al. 2014) although definitive conclusions generated by systematic reviews and meta-analysis could not be obtained concerning the clinical efficacy of chemomachanical caries removal system Carisolv (Marquezan et al. 2006; Li et al. 2014; Lai et al. 2015).

At present, there is also significant interest in the application of laser technology for removal of caries. Consequently erbium:yttrium-aluminium-garnet (Er:YAG) and erbium,chromium:yttrium-scandium-gallium-garnet (Er,Cr:YSGG) lasers have found favour for application on dental hard tissues (Shahabi and Zendede 2010; Schwass et al. 2013). Light emitted by Er:YAG lasers is strongly absorbed by water, resulting in rapid and expansive vapourisation of water in dentin, causing explosive dislocation of dental hard tissue components (de Almeida Neves et al. 2011; Schwass et al. 2013). Although there is limited evidence to support the clinical effectiveness of lasers in the removal of caries compared with conventional methods (Jacobsen et al. 2011), the authors concluded that erbium lasers (Er:YAG and Er,Cr:YSGG) are as effective as a rotary bur and adult patients preferred the laser over the rotary bur because the need for local anesthesia is lower due to the absence of vibration and a lower pain sensation (de Almeida Neves et al. 2011; Jacobsen et al. 2011). In spite of the favourable properties of erbium lasers, other evaluations regarding microindentation hardness and compositional changes of enamel and dentin irradiated with these lasers should be clarified before they can be routinely used as a caries removal method.

Limited in vitro studies comparing these two complemantary methods of caries excavation (chemomechanical and erbium laser techniques) have been performed so far (de Almeida Neves et al. 2011; Kinoshita et al. 2003), especially considering their effects on the features of the remaining dentin after caries removal in permanent teeth with natural occlusal dentin carious lesions. Therefore, the aims of this in vitro study were to investigate the Carisolv chemomechanical and Er:YAG laser caries excavation methods in comparison with the conventional tungsten-carbide bur excavation with regard to the extent of removing carious dentin based on the stereomicroscopic observations and microindentation hardness measurement of the residual dentin left at the cavity bottom after caries excavation. The chemical composition of remaining dentin examined by a scanning electron microscopy–energy dispersive X-ray spectroscopy (SEM–EDX) has also been evaluated after caries removal.

## Methods

### Sample selection

This study was conducted with the approval of the Ethical Committee of Suleyman Demirel University Faculty of Medicine, Isparta. The patient consent was obtained for using their teeth for research purposes. Freshly extracted human permanent molar teeth stored up to 3 months in the 0.2 % sodium azide solution (Merck, Darmstadt, Germany) at room temperature were used in this study. All teeth were hand-scaled to remove tissue remnants and debris, cleaned with a pumice slurry using a rubber cup, and air-dried. After visual examination and careful probing, sixty-nine extracted teeth with carious occlusal surfaces of different severity were radiographed. All images were acquired using bitewing projection geometry. The radiographs (E-speed, Ceadent, Strängnäs, Swiss) were taken for each tooth using a dental X-ray unit (Trophy Radiology, Marne La Vallee, France) operating at 65 kVp, 10 mA; an exposure time of 0.25 s, a film holder (Endo-Bite posterior, KerrHawe, Bioggio, Switzerland), and a focus-object distance of 36 cm was used. The films were processed in an automatic processor (Periomat Plus, Dürr Dental, Bietigheim-Bissingen, Germany). The radiographs were examined under standardized conditions by one examiner and occlusal caries lesions were diagnosed according to a five-point scale proposed by Espelid et al. (1994). The teeth were categorized as ‘Grade 4’ in which carious lesions in the middle third of the dentin and included in this study.

### Caries removal

**Fig. 1:**
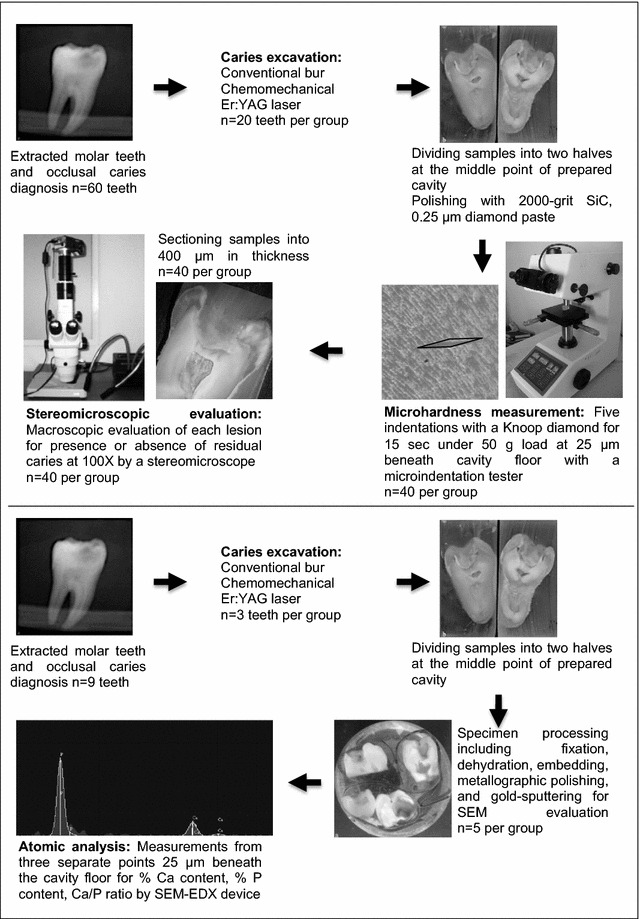
Three caries excavation groups of samples used in the study along with the evaluation methods performed

The occlusal cavities were prepared and unsupported enamel rods were removed using a cylindrical diamond bur (ISO 111.016, MDT Dental, Afula, Israel) with a highspeed handpiece and air/water spray (KaVo Dental, Biberach, Germany). The sixty teeth were randomly divided into three groups according to the three different caries removal methods. The experimental design is schematically shown in Fig. 1.

In the conventional caries removal method, the carious dentin tissue was mechanically removed using a round tungsten-carbide bur (H1SE ISO 016, Komet, Brasseler, Lemgo, Germany) mounted in a contra-angle slow-speed handpiece (micromotor and contra-angle, KaVo Dental, Biberach, Germany) with air as the coolant. Each bur was replaced with a new one after every five preparations. In the chemomechanical caries removal method, Carisolv system (Carisolv gel multimix, MediTeam Dental, Göteborg, Sweden) was used by using specifically designed hand instruments for this procedure. Carisolv gel was applied on the carious dentin surface for 30 s according to the manufacturer’s instructions, and the softened dentin was excavated with #2, #3 and #4 instruments. When the gel became cloudy it was rinsed with water and removed together with the dissolved carious dentin. Fresh gel was then applied and the cavity floor was repeatedly scraped until the gel became clear. For the laser caries removal technique, an Er:YAG laser (KaVo, Biberach, Germany) was applied with No: 2060 non-contact treatment (KaVo, Biberach, Germany) and in defocused mode under 15 ml/min water-cooling in accordance with the recommended parameters by the manufacturer (KaVo Key Laser 1243). The laser treated for removal of the carious dentin tissue had a 30.86 J/cm^2^, pulse energy 250 mJ and pulse frequency 4 Hz. The diameter of the laser beam treated from laser tip to dentin surface was 0.9 mm. The laser handpiece was mounted in a micro-manipulator (KITE-TB-L, World Precision Instruments, London, UK) to ensure that the laser beam was delivered perpendicular to the dentin surface at constant working distance of 10 mm.

Immediately after the caries removal, carious dentin tissue excavation was guided by the combined criteria of the tactile method and by a caries-staining dye in all groups. For the tactile examination hardness on probing were assessed. Dentin excavation was completed when hard dentin was detected using a dental probe (Banerjee et al. 2000). The caries-disclosing dye based on a solution of 1 % acid-red in propylene glycol (Caries Detector, Kuraray, Tokyo, Japan) was used according to the manufacturer’s instructions. The dentin, which was stained light pink in the cavity floor, was not removed in order to prevent excessive excavation.

### Microindentation hardness measurement

After excavation, the tooth samples were embedded in epoxy resin (Epofix kit, Struers, Denmark), and divided into two pieces in buccolingual direction at middle point of the prepared cavity using a slow-speed diamond saw (Micracut 125, Metkon, Bursa, Turkey) (n = 40 per group). The cut surfaces were polished with 2000-grit silicon carbide paper (Dempa P1, Metkon, Bursa, Turkey) and 0.25-μm particle size diamond pastes (Metadi II, Buehler, Illinois, USA) to create a flat smooth surface (Fig. 1).

The Knoop hardness (HK) value was measured for all the samples in a microindentation hardness tester (MH-3, Metkon, Bursa, Türkiye) with a 50 g load applied for 15 s at room temperature (23 °C). The indentations were made at the distance of 25 μm beneath the deepest point of the cavity floor. Five indentations at equal distances from each other were created on the flat surface of each specimen and each indentation on the surface was measured at 40× magnification. The average of the five readings was taken as the mean HK of the remaining dentin. The HK values were obtained directly from the digital readings on the screen of the tester.

### Stereomicroscopic evaluation

The specimens were sectioned longitudinally using a slow-speed diamond saw (Micracut 125, Metkon, Bursa, Turkey) and one section, approximately 0.4 mm in thickness, was obtained for each specimen (n = 40 per group). The microscopic evaluation was carried out at a magnification of 100× using a stereomicroscope (Olympus S2-STU 1, Japan) with an integrated digital camera (Olympus, Camedia C4000, Japan). The microscope images of the samples were transferred to a personal computer and stored as jpeg format for the visual macroscopic evaluation of each lesion (Fig. 1).

The presence or absence of residual caries were determined visually by two independent clinical examiner depending on the histological appearance of the areas. The darker brown areas corresponded to caries-infected dentin, the paler brown/translucent areas to caries-affected dentin and the yellow-white areas to the sound dentin (Almahdy et al. 2012). When disagreements occurred, examiners conducted a joint assessment to establish an agreement.

### Atomic analysis by SEM–EDX

Three teeth were prepared for each group in the same way for caries removal procedures and diagnosis of residual caries. After caries excavation was completed, the teeth were divided into two pieces in buccolingual direction starting at the middle point of the cavity (Fig. 1).

A total of five specimens for each group were randomly selected and were processed for scanning electron microscopy using specimen preparation techniques, including fixation in 2.5 % glutaraldehyde solution in cacodylate buffer solution and dehydration in ascending concentrations of ethanol (25, 50, 75, 95 and 100 %). The specimens were embedded in a chemically hardened epoxy resin. The epoxy molds were ground and polished with wet silicon carbide sandpapers of 800-, and 1200-grit size, and diamond polishing pastes of 3- and 1-μm grit-size. The specimens were then etched with 37.5 % orthophosphoric acid for 15 s, rinsed with water and air-dried in a desiccator. The samples were coated with 200 A° gold and examined by a SEM–EDX device (JEOL, JSM-6060, USA) at 20 kV accelerating voltage, title angle at 350° and 4000× magnification.

The measurements were obtained from three separate points at equal distances and 25 μm beneath the cavity floor. The obtained values of calcium (Ca) content, phosphorus (P) content and calcium/phosphorus (Ca/P) ratio were recorded in terms of percentage.

### Statistics

The microindentation hardness results were statistically evaluated by one-way ANOVA and Dunnett-C tests. The Kruskal–Wallis and Mann–Whitney U tests was used to test the significance of differences among the excavation techniques. Likewise, the comparisons between the methods for differences in calcium/phosphorus ratio of remaining dentin tissue were made using the Kruskal–Wallis and Mann–Whitney U tests. The statistical analyses were carried out using SPSS software version 10.0 (SPSS, Inc., Chicago, IL). In all tests the level of significance was set at 0.05.

## Results

Distribution of the number and percentages of residual caries for the three excavation methods are shown in Table 1. The conventional bur preparation and Er:YAG laser methods did not show any significant diference in number of residual caries (5 %) after stereomicroscopic evaluation (Kruskal–Wallis and Mann–Whitney U tests, p > 0.05; Table 1). However, the images after Carisolv chemomechanical caries removal method showed that the percentage of samples with remaining carious dentin (20 %) was higher in comparison with conventional and laser methods and the difference was statistically significant (Kruskal–Wallis and Mann–Whitney U tests, p < 0.05).

**Table 1:** Distribution of the number and percentages of residual caries for the three excavation methods (n = 40)

	Visual macroscopic evaluation by stereomicroscope
	Caries present/caries absent n (%)
Conventional	2/38 (5/95)^b^
Chemomechanical	8/32 (20/80)^c^
Er:YAG laser	2/38 (5/95)^b^

Mean values with the same superscript letters are not statistically different in the same column (Kruskal–Wallis and Mann–Whitney U test, p > 0.05)

**Fig. 2:**
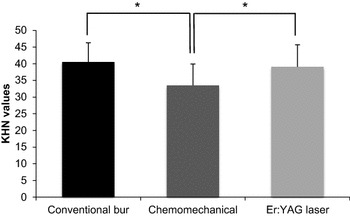
Mean microindentation hardness after caries removal. The values are expressed as the mean ± SD, *p < 0.05

The mean HK values of the samples for each group are presented in Table 2 and Fig. 2. There were no significant differences between the hardness values of remaining dentin at the lased and bur excavated cavity bottoms (One-way ANOVA, Dunnett-C test, p > 0.05). However, chemomechanical caries excavation result in a dentin substrate with a lower hardness (33.50 ± 6.39) when compared to conventional bur (40.51 ± 5.78), and laser excavation (39.09 ± 6.57; One-way ANOVA, Dunnett-C test, p < 0.05).

**Table 2:** Knoop hardness (HK) values of remaining dentin tissue after three caries removal methods

HK (mean ± SD) (n)		
Conventional	Chemomechanical	Er:YAG laser
40.51 ± 5.78^a^ (40)	33.50 ± 6.39^b^ (40)	39.09 ± 6.57^a^ (40)

Mean values with the same superscript letters are not statistically different (One-way ANOVA, Dunnett-C test, p > 0.05)

**Fig. 3:**
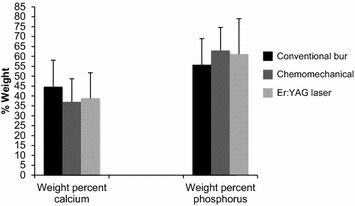
Comparison of the wt% of calcium and phosphorus for three caries removal methods. The values are expressed as the mean ± SD, p > 0.05

The mean quantities of Ca and P and Ca/P ratio are presented in Table 3 and Fig. 3. The mean of Ca (wt%) and P (wt%) were 44.68 ± 13.40; 55.76 ± 13.18, 37.03 ± 11.72; 62.95 ± 11.68, and 38.85 ± 12.87; 61.15 ± 17.87 for bur, Carisolv and Er:YAG laser treated surfaces, respectively. The Kruskal–Wallis and Mann–Whitney U tests revealed that there were no significant differences among the quantities of Ca (wt%) and P (wt%) in the conventional, Carisolv and Er:YAG laser groups (p > 0.05). In addition, no significant differences were found among the Ca/P ratio of the bur and chemomechanical excavated and lased cavities (p > 0.05).

**Table 3:** SEM–EDX values (mean ± SD) for each caries removal method (n = 5)

	Conventional	Chemomechanical	Er:YAG laser
Ca (wt%)	44.68 ± 13.40	37.03 ± 11.72	38.85 ± 12.87
P (wt%)	55.76 ± 13.18	62.95 ± 11.68	61.15 ± 17.87
Ca/P ratio	0.91 ± 0.50	0.86 ± 0.91	0.78 ± 0.54

No significant differences were found among the groups in terms of all criteria (p > 0.05)

## Discussion

When evaluating clinical operative techniques in in vitro studies, it has been stated that the use of natural dentin carious lesions should be considered instead of artificially-induced carious lesions (Banerjee et al. 2000). However, the influence of the variables such as the size, shape, depth and localization of natural carious lesions on the excavation results should be minimised (Banerjee et al. 2000; Dammaschke et al. 2005). In order to standardize these variables, only the permanent teeth with occlusal carious lesions into the middle third of the dentin detected on the radiograph were included in this study. Furthermore, by randomly dividing the teeth into three groups and by sectioning of the excavated teeth into two halves increase the number of specimens and minimise the excavation results.

Regarding minimal invasive tooth preparation, the endpoint of caries excavation can clinically be defined based on the hardness of dentin as felt with a dental probe, and also more subjective features such as the colour and moisture of the excavated dentin (de Almeida Neves et al. 2011; Neves Ade et al. 2011). In an attempt to develop an objective caries-removal procedure, the clinical use of staining agents were included (Fusayama 1993). The clinical and laboratory studies, however, revealed that hard and sound pulpal floors stain more easily because of its lower degree of mineralization and it has been recommended that light-pink stained tissue should be left in the cavity (Kidd et al. 1989; Yip et al. 1994). In this study, caries removal endpoint was reached when a ‘hard’ cavity floor was felt using dental explorer with a gentle pressure and the caries detector dye was used to guide and limit excavation to caries-infected dentin following excavation in all groups. However, residual dentin stained light-pink with a caries-staining dye retained in the cavity as instructed (de Almeida Neves et al. 2011; Neves Ade et al. 2011).

In addition to aforementioned clinical methods, laboratory methods including several microscopic techniques have also been described for the histological validation of caries-infected dentin during the in vitro comparison and evaluation of new minimally invasive excavation techniques. Because viewing hemisected teeth under a microscope is more accurate than visual examination of the intact tooth. To determine the true presence of caries, residual caries diagnosis has been performed under a stereomicroscope in this study (Ricketts et al. 1998). However, the stereomicroscopic evaluation was performed after the data from microindentation hardness testing were gathered because the specimens has to be initially cut with a saw and then gradually ground down to be able to examine histologically.

The presence or absence of residual dentin after caries removal is of great interest to clinicians and may affect decisions about the extent of carious dentin excavation. In this study, caries was defined as present when a darker brown discoloration was observed in the cavity floor using stereomicroscopic images. It is very difficult to give accurate guidelines for sound and hard dentin differentiation. This issue is very prone to subjectivity and very dependent on the operator’s perception and experience (Celiberti et al. 2006). That’s why the photographs obtained using a stereomicroscope for the visual macroscopic evaluation of each lesion and the macroradiographs were subjectively assessed by two examiners.

Carisolv is a sodium-hypochlorite-based dentin solubilising agent used to selectively dissolve carious dentin and the extent of carious dentin excavation is based on the self-limiting capacity of the solution. The efficacy of Carisolv and conventional hand excavation in establishing the end-point of caries excavation by removing carious dentin was found similar extent when related to the auto-fluorescence signature of carious tissue detected by confocal microscopy (Banerjee et al. 2000). However, an in vitro study has shown that the Carisolv treatment resulted in higher mean depths of the remaining caries-active lesion in dentin than conventional caries removal with round bur, as determined in microscopic images (Splieth et al. 2001).

The effectiveness of carious dentin removal with erbium lasers irrespective of the parameters used during laser irradiation has also been questioned over the years. Controlled caries excavation becomes more difficult due to the lack of tactile sensation because a noncontact beam emission mode is recommended for optimal irradiation when using erbium lasers (de Almeida Neves et al. 2011; Celiberti et al. 2006). Moreover, irregularity of the dentin surfaces left after laser ablation hampers an adequate tactile feedback for proper clinical hardness assessment with the probe (de Almeida Neves et al. 2011; Celiberti et al. 2006). Despite these factors, favourable results were recorded for a non-contact Er:YAG laser used in this study and the number of cavity with residual dentin did not differ significantly between the conventional bur and laser excavation techniques, while the chemomechanical excavation presented to leave more carious dentin behind. The results of this study are in agreement with the comparative study from Kinoshita et al. (2003). In their study light microscopic observations have also disclosed better scores of residual dentin after excavation with an Er,Cr:YSGG laser than with Carisolv.

Microindentation hardness testing has been used in in vitro studies to determine the hardness of different carious layers and is commonly associated with the relative mineral content of dental hard tissues (Almahdy et al. 2012). This study compared the microindentation hardness to the visual macroscopic appearance of remaining dentin left at the cavity bottom after three caries excavation techniques. The microindentation hardness value of residual dentin left at the cavity bottom after Carisolv excavation was significantly lower than those obtained after conventional and laser excavations, as previously reported (Hamama et al. 2013; Magalhães et al. 2006). Carisolv was reported to leave more demineralised (caries-affected) dentin on the cavity floor, which may be a possible explanation for the decreased microindentation hardness measured in this study (Hamama et al. 2013; Magalhães et al. 2006). According to atomic analysis, the calcium and phosphorus content remain similar after all excavation methods, which is also in agreement with another study (Hamama et al. 2013). They also compared conventional bur guided by a caries-staining dye and Carisolv, as was used in this study. Moreover, a similar Ca (wt%) and P (wt%) or Ca/P ratio, indicating no chemically-induced changes of dentinal components, were found with Carisolv cavities as compared to adjacent sound dentin areas used as a control reference (Hossain et al. 2003). The results of our study confirmed that previous study about on the possible use of Carisolv caries removal system when a proper clinical guide is used in clinical dentistry.

Although in literature a limited data is available regarding residual dentin hardness obtained for caries-affected dentin after laser ablation, in most of the studies, extracted noncarious teeth have been used to compare the remaining dentin microindentation hardness and structural changes in the cavity floor prepared by erbium lasers and conventional rotary instruments (Shahabi and Zendede 2010; Hossain et al. 2003; Souza-Gabriel et al. 2009; Chinelatti et al. 2010; Al-Omari and Palamara 2013). No significant alterations were demonstrated for the chemical composition and microstructure of dentin after excavation with erbium lasers as compared to conventional tungsten-carbide bur, using microindentation hardness testing and energy dispersive X-ray spectroscopy (Shahabi and Zendede 2010; Hossain et al. 2003; Souza-Gabriel et al. 2009). The microindentation hardness measurement and SEM–EDX have disclosed the same degree of hardness value and Ca/P ratio of residual dentin left at the cavity bottom by an Er:YAG laser and conventional bur excavation for carious teeth used in this study, which is also in agreement with these studies.

Er:YAG and Er,Cr:YSGG laser irradiation previously revealed a significant increase in the quantities of Ca (Ca wt%) and P (P wt%) in the prepared cavities (Hossain et al. 2002, 2003). These changes have been described to the result from evaporation of organic components of the tissue because of an increase in temperature in the irradiated area (Shahabi and Zendede 2010). However, minimal heat-induced changes in dentine components after Er:YAG irradiation with a water spray by cooling the irradiated area and absorbing excessive laser energy can be achieved, as was confirmed by the SEM–EDX results without significant difference among the compositional structure (the Ca wt%, P [P wt% and Ca/P ratio) of the laser- and bur-treated cavities in this study (Shahabi and Zendede 2010).

Regarding the laser settings most commonly advised for cavity preparation, the most important parameters are energy levels and pulse repetition rate because they are related to the laser’s ablation ability and to the deposition of residual heat on dental substrates (Souza-Gabriel et al. 2009). Use of the lower parameters (200–300 mJ, 2 Hz) for preparation with the Er:YAG laser produced dentin microindentation hardness results similar to those for bur-prepared cavities (Souza-Gabriel et al. 2009). Similarly, Chinelatti et al. (2010) have shown that high Er:YAG laser energy levels (260–360 mJ, 3 Hz) reduced the subsurface microindentation hardness of superficial and deep dentin because of the denaturation of the dentin organic matrix with a strong modification of the collagen chain. In this study, microindentation hardness measurement was performed under cavity preparations at distances of 25 μm from the middle of the cavity floor (superficial dentin) with Er:YAG laser irradiation used at low power settings (250 mJ/4 Hz), which may be a possible explanation of the similar microindentation hardness measurement for laser- and bur-prepared dentin.

In addition to the traditional residual caries detection methods such as histology, high-technology research techniques with quantitative data analysis have currently been investigated, such as micro-computerized tomography to compare caries excavation techniques (Schwass et al. 2013; Neves Ade et al. 2011; Zhang et al. 2013). In previous research, although Er:YAG laser ablation could not be considered a technique for selectively remove caries-infected dentin, leaving residual dentin with an acceptably high mineral density (Neves Ade et al. 2011), the potential of the diode pumped Er:YAG laser operating at high pulse repetition rates for the selectively remove areas of demineralization has recently been demonstrated (Yan et al. 2015). Systematic studies with high-technology research techniques should be performed to compare and clarify the effects of different and newer caries removal methods on selective removal of carious tissue, and also cavity morphology and the features of the residual dentin after caries removal.

The results indicated that Er:YAG laser ablation was more comparable to conventional bur excavation than chemomechanical method in the efficacy of caries removal with regard to microindentation hardness of remaining dentin and both chemomechanical caries removal agent Carisolv and Er:YAG laser did not alter chemical composition of residual dentin in the treated cavities.

## Abbreviations

Er:YAG: erbium:yttrium-aluminium-garnet
Er,Cr:YSGG: erbium,chromium:yttrium-scandium-gallium-garnet
SEM–EDX: scanning electron microscopy–energy dispersive X-ray spectroscopy
Ca: calcium
P: phosphorus
